# Timing of Maternal Stress Differentially Affects Immune and Stress Phenotypes in Progeny

**DOI:** 10.3390/ani14213074

**Published:** 2024-10-25

**Authors:** Cassidy Reddout-Beam, Lily P. Hernandez, Janeen L. Salak-Johnson

**Affiliations:** Department of Animal and Food Sciences, Oklahoma State University, Stillwater, OK 74078, USA; cassidy.reddout@usda.gov (C.R.-B.); lily.hernandez@okstate.edu (L.P.H.)

**Keywords:** stress, immune, maternal influence, gestational stress, passive immunity

## Abstract

Maternal stress during gestation may affect the development and responsiveness of the hypothalamic–pituitary–adrenal axis (HPA) and immune system in the progeny. Stressor type, duration and gestational stage at which the stressor occurs may all influence the short and long-term effects on the future progeny. The present study advances the characterization of the timing of gestational stress on the stress responsiveness and immune and behavioral phenotypes of the progeny. First, parity sows were hand-fed hydrocortisone acetate (stressed) or placebo (controls) during mid or late gestation. Colostrum and cord blood were collected. Blood samples were obtained from a subset of piglets (n = 36) born to these sows during lactation, pre- and post-weaning, and during an ACTH challenge. Stress and immune measures were assessed. Piglets born to sows stressed during mid-gestation had reduced measures of humoral immunity, including immunoglobulins and interleukin-4. Conversely, piglets born to late-stressed sows exhibited a delayed or dampened stress response to weaning and an ACTH challenge. They also had a skewed pro-inflammatory phenotype, as evidenced by increased interleukin-17 and tumor necrosis factor-α. Overall, these data suggest that the stage of gestation at which gestational stress occurs has differential effects on the HPA axis and immune development of the progeny, resulting in differential stress responsiveness and immune and behavioral phenotypes.

## Simple Summary

The present study aimed to detail the stress responsiveness and immune and behavioral phenotypes of progeny born to chronically stressed sows during mid or late gestation. Sows stressed during mid-gestation had reduced immunoglobulins in their colostrum, and their piglets had lower measures of humoral immunity. At the same time, piglets born to sows stressed during late gestation had altered stress response and behavior. These results indicate that the timing of gestational stress has a differential impact on the stress axis and immune development of the progeny at birth and up to 21 days post-weaning.

## 1. Introduction

Maternal stress can expose the developing fetus to high glucocorticoid levels by crossing the placental barrier, resulting in lifelong effects on physiology, immune function, and behavior in the progeny [1,2]. More specifically, exposure during fetal development can affect the hypothalamic–pituitary–adrenal (HPA) axis, resulting in postnatal changes in behavior and stress responsiveness of the progeny [3,4]. In pigs, high maternal cortisol exposure can induce postnatal changes in the corticotropic axis, indicating that prenatal stress effects may be partly due to changes in this axis [5,6]; however, placental insufficiency may also contribute to developmental changes [7]. Maternal immunity and postnatal care may be affected, which may also impact the physiology and behavior of the progeny postnatally [8]. However, prenatal stress does not always affect the progeny’s physiology, behavior and performance in the postnatal period, which may be partly due to the gestational period in which maternal stress occurs and the stress paradigm used [3].

Different maternal stress models that simulate housing or management situations or endogenously elevate maternal cortisol, such as repeated adrenocorticotropic hormone (ACTH) injections or administering oral hydrocortisone acetate (HCA), can cause developmental, HPA regulation, behavioral and immunological changes [3]. Stress responsiveness was increased among progeny born to sows subjected to social or ACTH-induced stress during mid or late gestation [9,10] but not early gestation [11]. Interestingly, HPA regulation is altered when HCA is administered during late, not mid-gestation. Stress-related behaviors, including escape and vocalization, were increased in piglets from sows receiving HCA or ACTH during late gestation [12,13]. However, these behaviors were not altered among those born to sows challenged with ACTH during mid-gestation [14].

Moreover, the gestational period in which maternal stress occurs can also affect the ontogeny and maturation of the immune system of the progeny. One critical window of vulnerability in pigs especially sensitive to maternal stress is the myeloid period, in which the lymphocytic populations rapidly expand between gestational days 60 and 80 [15]. Progeny born to sows that were subjected to restraint or social stress or administered ACTH during late gestation exhibited altered lymphocyte proliferation and reduced immunoglobulin levels and leukocyte populations, but there was no effect on progeny born to non-stressed sows [16] or sows experiencing these stressors during early [11] or mid-gestation [13]. It is plausible that maternal cortisol may directly affect fetal immune development, as cortisol can pass through the placental barrier [3] or indirectly affect the immune response of the progeny.

It is plausible that cortisol may disrupt the necessary shift in a Th-1 (cell-mediated) to Th-2 (humoral) phenotype at the maternal-fetal barrier [17]. Glucocorticoids drive the Th-1 and Th-2 immune bias [18], thus altering the immune profile in utero, translating to an altered immune profile postnatally [19]. Another possibility is that cortisol-related effects on the immune response of the dam may affect passive immunity via changes in the immune composition of colostrum and milk, as maternal immunoglobulins were reduced in the colostrum of sows subjected to heat stress in late gestation [20]. Reduced immune factors in the colostrum and milk may affect the immune development of the progeny. This is especially a concern in species such as swine, where maternal immune cells, immunoglobulins and other large molecules do not cross the placental barrier [15,21], making them more vulnerable due to being dependent on colostrum for immunoprotection.

Prenatal stress can affect immunological development and immune function in the progeny and thereby alter the disease susceptibility; however, the outcome may be dependent upon maternal stressor, gestational period, and the experiences of the progeny postnatally. The link between maternal prenatal stress at different stages of gestation on the HPA axis and the immune system of the progeny postnatally is relatively limited. Thus, the current study employed a pharmacological model that mimics prenatal stress in pregnant sows by increasing circulating cortisol concentrations in a controlled manner [12,22] via oral administration of hydrocortisone acetate (HCA), a synthetic glucocorticoid, to primiparous sows during mid and late gestation to characterize the stress responsiveness and immune and behavioral phenotypes of the progeny by measuring cortisol, leukocyte populations, cytokines, immunoglobulins and agonistic and oral-nasal-facial (ONF) behaviors. Determining if gestational stress and the period at which stress occurs alter stress and immune measures in the progeny may highlight important time points to minimize maternal stress to maximize the well-being of the progeny.

## 2. Materials and Methods

All animal procedures were approved by the Institutional Animal Care and Use Committee at Oklahoma State University under protocol no. IACUC-20-19.

### 2.1. Animals and Experimental Design

A total of 10 Yorkshire × Landrace cross primiparous gilts were artificially inseminated using a single Landrace semen source (DNA Genetics, Columbus, NE, USA). Gilts were kept in standard gestation stalls (0.56 m × 2.36 m) within a mechanically ventilated gestation house at Oklahoma State University Swine Research and Teaching Facility (Stillwater, OK, USA). Pregnancy was confirmed ultrasonically at 38 ± 2 days post-breeding. Gilts were randomly assigned to receive either hydrocortisone acetate capsules (HCA; Spectrum, New Brunswick, NJ, USA), a synthetic glucocorticoid or placebo during gestational days 51 through 72 (mid-gestation; M-HCA n = 3 or M-CON n = 2) or 81 through 102 (late gestation; L-HCA n = 3 or L-CON n = 2). The gilts were hand-fed one HCA gelatin capsule (70 mg/capsule) or placebo (empty gelatin capsule) at 0600 h and another at 1630 h for 21 days. At gestational day 110, all animals were moved and kept in individual farrowing stalls until the end of a 21-day lactation period.

To minimize potential piglet variation due to birth order or uterine placement, during the farrowing process, the first, fourth, and eighth piglets born to each sow were weighed and ear-tagged for identification. The last piglet born that allowed us to balance for sex was weighed and tagged for 4 piglets per litter, resulting in a subset of 36 piglets being used in this study. This subset of piglets remained with their sow and littermates until they were weaned at 21 ± 2 days old. Body weights were recorded at 7, 14, and 21 d post-birth by placing each piglet individually on a digital bucket scale.

Once piglets were weaned, they were moved to a new mechanically ventilated nursery building where they were randomly allotted to same-sex pens balanced for body weight across treatments; thus, each pen housed 2 piglets/treatment from different sows, but the same sex (n = 6 piglets per pen with each treatment be represented). Each pen (n = 6 pens) contained one nipple waterer, and they were fed ad libitum, a standard nursery diet formulated to meet or exceed NRC requirements [23]. At 42 days of age (or 21 days post-weaning), a subset of piglets were randomly selected and injected intramuscularly with 222 µg of ACTH dissolved in 1 mL of saline (n = 12) or 1 mL of saline (n = 12). Body weights were recorded at 7, 14, and 21 d post-weaning by placing each piglet individually on a digital bucket scale.

### 2.2. Sample Collection

Colostrum was collected from each sow after the onset of the birthing process but prior to the delivery of the first piglet. Samples were collected by placing two fingers and stripping at least 0.5 mL per teat until at least 5 mL of colostrum was collected from each functional teat. Samples were placed on ice, aliquoted, and stored at −80 °C until further analysis.

Umbilical cord blood was collected prior to suckling from a subsample of piglets at birth. The umbilical cord was placed between two fingers, stripping blood into a vacutainer containing heparin. Blood samples were collected at 7, 14, and 21 days of age, at 24 h and 7 days post-wean, and then prior to ACTH challenge and 1, 2, 4, and 24 h post-challenge. Piglets were held supine, and samples were collected via jugular venipuncture using heparin vacutainers (procedure lasted < 2 min; B.D. Vacutainers; Franklin Lakes, NJ, USA). Samples were immediately put on ice until processing could be completed.

### 2.3. Complete Blood Cell Count and Lymphocyte Isolation and Proliferation Assay

An aliquot of whole blood was added to an Eppendorf tube for a complete blood cell count (CBC), which was determined electronically using the Element HT5 Hematology Analyzer (Heska, Loveland, CO, USA). Vacutainers were centrifuged at 2100 rpm for 25 min. The plasma was aliquoted and stored at −20 °C until further analysis.

Whole blood was diluted 1:1 with Roswell Park Memorial Institute (RPMI; Gibco, Carlsbad, CA, USA) medium, layered over Histopaque-1077 (density = 1.077 g/mL; Sigma, St. Louis, MO, USA) and centrifuged at 700× *g* for 30 min at 25 °C. Lymphocytes were then removed, washed twice in RPMI and counted. Cell concentrations were then adjusted to 5 × 10^6^ cells/mL with a solution of 90% RPMI with 10% fetal bovine serum (R10) and placed into the wells of a sterile 96-well flat-bottom plate. Concanavalin A (ConA) and lipopolysaccharide (LPS) were used as mitogens (Sigma, St. Louis, MO, USA) to stimulate T and B cells, respectively, at a concentration of 0, 0.2, 2.0, and 20 μg/mL for ConA and 0, 0.5, 5.0 and 50 μg/mL for LPS. Mitogen concentrations were pipetted into the 96-well plates in triplicate. Then, the plates were incubated for 48 h at 37 °C in a 5% CO_2_-humidified incubator. From the top of each well, 100 μL was removed and R10 was added; then, the plates were returned to incubation for 18 h. Then, 20 μL of CellTiter 96^®^ AQueous One Solution Reagent (Promega, Madison, WI, USA) was added to each well and the plates were returned to incubation for 4 h. Plates were read using a microplate reader (BioTek Instruments, Winooski, VT, USA) at 490 nm with a reference wavelength of 690 nm. Results are expressed as a proliferation index (P.I.): Optical Density (490/690 nm) stimulated cells–Optical Density (490/690 nm) non-stimulated cells.

### 2.4. Cortisol, Cytokines, Stress Markers, and Immunoglobulins

Cortisol, cytokine, stress marker, and immunoglobulin concentrations were all measured using a commercially available enzyme-linked immunoassay (ELISA) following the manufacturer’s protocol with minor modifications. Cortisol was measured using kits from Arbor Assays (Ann Arbor, MI, USA) by diluting plasma samples 1:100 and colostrum 1:200. Interleukin-4, -10, and -17, interferon-γ (IFN-γ), and tumor necrosis factor-α (TNF-α) were measured using swine-specific ELISA kits from Invitrogen Corp. (Waltham, MA, USA) by diluting plasma samples 1:2 and colostrum 1:10 for IL-4, INF-ɣ, and TNF-α, and at 1:20 for IL-10 and IL-17. Immunoglobulin G (IgG) and A (IgA) were measured using swine-specific ELISA kits from Bethyl Laboratories Inc. (Montgomery, TX, USA) by diluting plasma samples 1:500,000 or 1:10,000 (IgG and IgA, respectively) and colostrum samples 1:1,000,000 or 1:30,000 (IgG and IgA, respectively). Immunoglobulin M (IgM) was measured using swine-specific ELISA kits from Abnova (Taipei City, Taiwan) by diluting colostrum samples 1:20,000. Immunoglobulin E (IgE) was measured using swine-specific ELISA kits from BIOTANG Inc. (Lexington, MA, USA) by diluting colostrum samples 1:20. Corticosteroid-Binding Globulin (CBG) and 11β-Hydroxysteroid dehydrogenase-2 (11β-HSD2) were measured using swine-specific ELISA kits from BlueGene Biotech (Shanghai, China) by using undiluted plasma samples for CBG and diluting plasma samples 1:2 for 11β-HSD2. Corticotropin-releasing hormone (CRH) and Adrenocorticotropic Hormone (ACTH) were measured using swine-specific ELISA kits from FineTest Biotech Inc. (Boulder, CO, USA) by running undiluted plasma samples for CRH and diluting plasma samples 1:5 for ACTH. All samples were run in duplicates.

Plates were read at a wavelength of 450 nm, and a standard curve was generated using a microplate reader (BioTek Epoch; Gen5 Version 3.04.17 Data Analysis Software; Bio-Tek, Winooski, VT, USA) to determine the concentration of the unknown samples. The minimal detectable concentration for cortisol was 27.6 pg/mL, and for Interleukin-4, -10, -17, Interferon-gamma, and Tumor Necrosis Factor-α it was 1.5 pg/mL, 3.0 pg/mL, 14 pg/mL, 2.0 pg/mL and 3.0 pg/mL, respectively. The minimal detectable concentration for IgG and IgA was 1.37 ng/mL. Lastly, the minimal detectable concentration for CBG, 11β-HSD2, CRH, and ACTH was 1.0 ng/mL, 1.0 ng/mL, 1.6 pg/mL, and 12.5 pg/mL, respectively.

### 2.5. Behavioral Observations

Behavior was captured using color surveillance cameras (Night Owl SP, Boca Raton, FL, USA). Cameras were mounted on the ceiling and positioned over the pen to video record all 6 piglets per pen. Continuous sampling was used to observe and register behavior for 4 h post-weaning and mixing (n = 36 piglets; 6 piglets/pen). Both frequency and duration of agonistic and oral-nasal-facial (ONF) behaviors were registered. The ONF behaviors were further classified as animate (toward conspecific) or inanimate objects (floor, feeder, bars, waterer).

### 2.6. Statistical Analysis

Data were analyzed utilizing the correlation and mixed model with repeated measures in SAS 9.4 (Inst. Inc., Cary, NC, USA). All traits were tested for departure from a normal distribution through analysis of the residuals; therefore, log transformation was applied to these variables: cortisol, 11β-HSD2, CBG, CRH, ACTH, immunoglobulin G and A, and cytokines. This model included fixed effects of maternal cortisol treatment (HCA or CORT) and gestational period (MID or LATE) for the sow and fixed effects of sow treatment (M-HCA, L-HCA or CON) for farrowing characteristics, colostrum and piglet measures. Significance was set at (*p*-value ≤ 0.05) with trends discussed at (*p*-value > 0.05) to (*p* ≤ 0.10). The Tukey–Kramer adjusted *p*-value was utilized to analyze farrowing characteristics, colostrum and piglet measures to control for Type 1 Error.

## 3. Results

### 3.1. Maternal Treatment Effects at Birth

Colostrum and umbilical cord blood samples were characterized to determine maternal treatment effects. Colostral IgA concentrations were higher (*p*-value = 0.01) in the CON and L-HCA-treated sows than in the M-HCA-treated ones, and colostral IgG, IgM, IgE concentrations, cytokines, and cortisol were similar across treatment groups (Table 1).

Umbilical cord blood from piglets born to L-HCA-treated sows had higher percentages of neutrophils (*p*-value < 0.01) and lower percentages of lymphocytes (*p*-value = 0.01) than those from M-HCA-treated ones and higher (*p*-value = 0.02) cortisol than piglets from sows in other treatments (Table 2). This shift in neutrophil and lymphocyte percentages among these piglets also resulted in a higher (*p*-value = 0.01) N:L ratio than those from M-HCA-treated sows (Table 2). No other measures, including IgG, IgA, IL-10, TNF-α, IFN-ɣ, CRH, ACTH, and 11-βHSD2 concentrations measured in umbilical cord samples, were different amongst all treatment groups. The average birth weight of the selected piglets did not differ based on sow treatment (M-HCA = 1.20 kg, L-HCA = 1.49 kg and CON = 1.45 kg; *p*-value = 0.14).

### 3.2. Maternal Prenatal Stress on Progeny During Suckling Period

**Figure 1:**
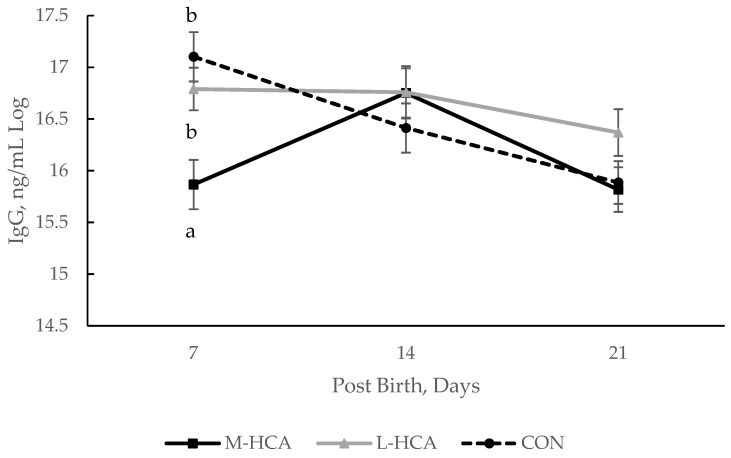
Immunoglobulin-G concentrations for piglets over a 21-day lactation period were born to maternally stressed sows, n = 36. Sow treatments were hydrocortisone acetate (HCA) capsules or placebo (CON) fed for 21 days during mid-gestation (M-HCA) or late gestation (L-HCA). Data are expressed as means ± the standard error of the mean. ^a,b^ Means with different superscripts differ at *p*-value < 0.05 between treatments within a day according to the Tukey–Kramer adjustment.

**Figure 2:**
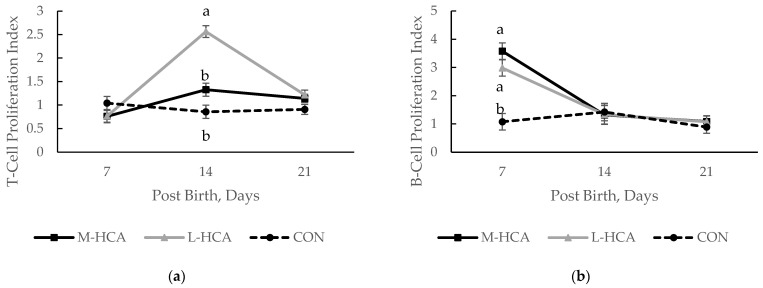
Effect of maternal treatment on T-cell proliferation response to concanavalin A (**a**) and B-cell proliferation response to LPS (**b**) in the piglet over the 21d lactation phase, n = 36. Sow treatments were hydrocortisone acetate (HCA) capsules or placebo (CON) fed for 21 days during mid-gestation (M-HCA) or late gestation (L-HCA). Data are expressed as means ± the standard error of the mean. ^a,b^ Means with different superscripts differ at *p*-value < 0.05 between treatments within day. Treatment x Day *p*-value < 0.01 for both according to the Tukey–Kramer adjustment.

During the 21-day suckling period, an interactive effect of sow treatment and days post-birth occurred for plasma IgG concentration (*p*-value = 0.02; Figure 1) and mitogen-induced T- and B-cell lymphocyte proliferation indexes (*p*-value < 0.01; Figure 2). Differing patterns of total IgG concentrations were found between piglets from M-HCA and L-HCA-treated sows, with IgG concentrations being lower among piglets born to M-HCA-treated ones at D7 and then higher at D14 post-birth compared with those piglets born to L-HCA-treated or CON sows (Figure 1). At the same time, IgG peaked at D7 post-birth and then declined at D14 and D21 among piglets from CON sows. Conversely, IgG concentrations remained elevated and relatively constant among piglets from L-HCA-treated sows throughout the lactation period (Figure 1).

The mitogen-induced T-cell lymphocyte proliferation index was also affected across the 21-day lactation period, with those piglets born to L-HCA-treated sows having the highest index, while those piglets from CON sows had the lowest index at 14D post-birth (Figure 2a). The mitogen-induced B-cell lymphocyte proliferation index was highest in piglets from M-HCA-treated sows and lowest in those from CON ones at D7 post-birth (Figure 2b). Interestingly, these piglets had the highest B-cell proliferation and the lowest IgG concentrations, which is the opposite of what one would expect since B-cells are associated with humoral immunity.

Overall, sow treatment also tended to affect other measures, including neutrophil percentage (*p*-value = 0.09), N:L ratio (*p*-value = 0.09), and IgA (*p*-value = 0.08). Similarly to umbilical cord blood analysis, mean neutrophil percentages and N:L ratio were highest in piglets from L-HCA-treated sows and lowest in those from M-HCA-treated (Table 3). In contrast to umbilical cord blood, but similar to colostrum, mean IgA concentrations were lowest in piglets from M-HCA-treated sows and highest amongst those from CON ones (Table 3). Average weight at weaning did not differ based on sow treatment (M-HCA = 5.13 kg, L-HCA = 5.89 kg and CON = 5.39 kg; *p*-value = 0.97).

### 3.3. Prenatal Stress and Wean and Mix Stressors: Immune and Cortisol Measures

**Figure 3:**
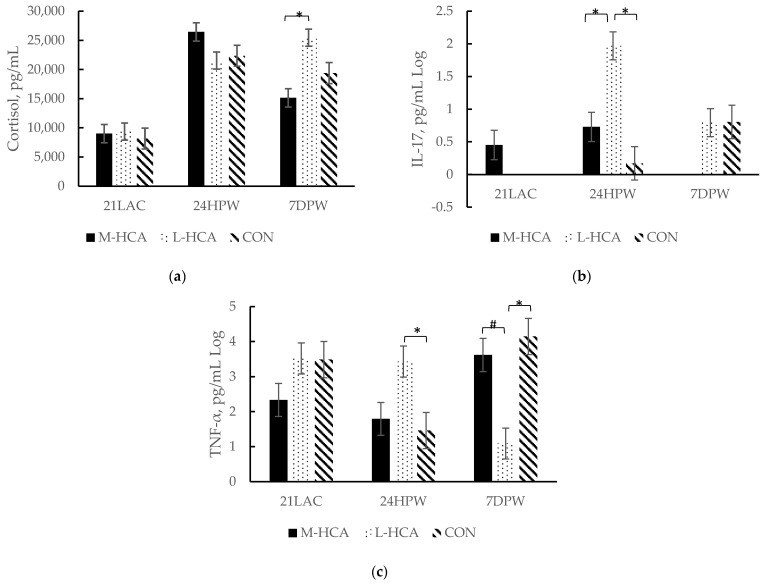
Plasma cortisol (**a**), interleukin-17 [IL-17] (**b**) and tumor necrosis factor-α [TNF-α] (**c**) concentrations in prenatally stressed piglets (n = 36) at 21 days of lactation [21LAC], and 24 h [24HPW] and 7 days [7DPW] post-weaning. Sow treatments were hydrocortisone acetate (HCA) capsules or placebo (CON) fed for 21 days during mid-gestation (M-HCA) or late gestation (L-HCA). Data are expressed as means ± standard error of the mean. Means with an * differ at *p*-value < 0.05 and # at 0.05 < *p*-value < 0.10 between treatments according to the Tukey–Kramer adjustment. Treatment x Day *p*-value = 0.09 for cortisol, *p*-value = 0.04 for IL-17, and *p*-value = 0.02 for TNF-α.

Plasma cortisol (*p*-value = 0.09), IL-17 (*p*-value = 0.04), TNF-α (*p*-value = 0.02) and mitogen-induced T-cell lymphocyte proliferation indexes (*p* = 0.01) were affected by maternal treatment, with those from M-HCA-treated and CON sows having similar concentrations and those from L-HCA-treated ones differing in response to weaning. Specifically, cortisol concentrations peaked 24 h post-weaning among piglets from M-HCA-treated sows and CON ones (Figure 3). The cortisol response to weaning stress did not peak until 7 days post-weaning among those piglets from the L-HCA-treated sows. These piglets also had higher concentrations of pro-inflammatory cytokines, including IL-17 and TNF-α, at 24 h post-weaning than piglets from M-HCA or CON dams (Figure 3). Conversely, TNF-α concentrations peaked at 7D post-weaning among those piglets from the M-HCA-treated and CON sows. However, no effect on IL-17 was observed, but it should be noted that IL-17 may be involved in monocyte recruitment. Piglets from L-HCA-treated sows also had higher monocyte percentages in the post-wean phase (M-HCA = 7.07%, L-HCA = 8.10% and CON = 7.99%; *p*-value = 0.04). Moreover, anti-inflammatory IL-4 concentrations were lowest overall for piglets from M-HCA-treated sows in the post-wean phase than piglets from L-HCA or CON sows (*p*-value = 0.03).

**Figure 4:**
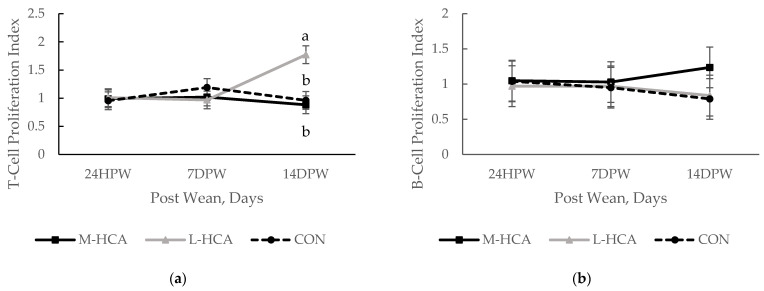
Effect of maternal treatment on T-cell proliferation response to concanavalin A (**a**) and B-cell proliferation response to LPS (**b**) in the piglets during the post-wean phase, n = 36. Sow treatments were hydrocortisone acetate (HCA) capsules or placebo (CON) fed for 21 days during mid-gestation (M-HCA) or late gestation (L-HCA). Data are expressed as means ± the standard error of the mean. ^a,b^ Means denoted with different superscripts differ at *p*-value < 0.05 between treatments within day, according to the Tukey–Kramer adjustment. Treatment x Day *p* = 0.01 for T-cell proliferation and *p*-value = 0.91 for B-cell proliferation.

As observed in the lactation phase, the mitogen-induced T-cell lymphocyte proliferation index was highest in piglets from the L-HCA-treated sows at 14 days post-weaning (Figure 4). However, no other effects on lymphocyte proliferation were noted, nor were cytokines or leukocyte populations affected by maternal treatment in the post-wean phase.

### 3.4. Prenatal Stress and Wean and Mix Stressors: Behavior and Weight

Maternal treatment did not affect the frequency or duration of aggressive encounters, nor did sow treatment significantly influence which piglet was likelier to initiate or receive aggressive acts at mixing (Table 4). While not significant, it is interesting to note that piglets from L-HCA-treated sows did spend the least time engaged in an aggressive encounter, especially if they initiated the encounter. These piglets were also found to be the initiator or receiver of aggressive encounters less often than their counterparts from M-HCA-treated or CON sows, reducing the time spent engaged in aggression. Maternal treatment did, however, affect the frequency and duration (*p* = 0.04 and *p* = 0.07, respectively) of overall oral-nasal-facial (ONF) stereotypic behavior (Table 4), which was reduced in piglets from L-HCA-treated sows, with these piglets performing ONF behaviors 119% and 113% less often than those from M-HCA-treated and CON sows, respectively. Moreover, piglets from L-HCA-treated sows spent 113% less time performing these behaviors than their counterparts from M-HCA-treated sows.

**Figure 5:**
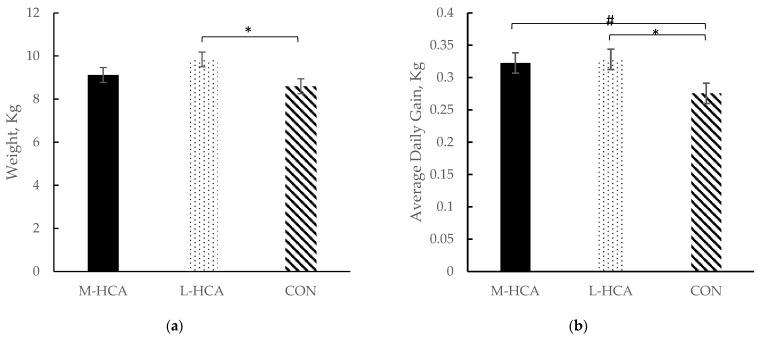
Effect of maternal treatment on body weight (**a**) and average daily gain (ADG) (**b**) in piglets overall for the post-wean phase, n = 36. Sow treatments were hydrocortisone acetate (HCA) capsules or placebo (CON) fed for 21 days during mid-gestation (M-HCA) or late gestation (L-HCA). Data are expressed as means ± standard error of the mean. Means with a * differ at *p*-value < 0.05, and means with a # between treatments differ at 0.05 < *p*-value < 0.10 according to the Tukey–Kramer adjustment. Treatment effect *p*-value = 0.04 for weight and *p*-value = 0.04 for ADG.

While piglets from L-HCA-treated sows were the heaviest overall (*p* = 0.04), piglets from sows administered HCA, regardless of stage of gestation, exhibited higher average daily gains (*p* = 0.04) than piglets from CON sows overall for the post-wean phase (Figure 5). Piglets from L-HCA-treated sows were 14% heavier than those from CON sows (Figure 5a). Also, ADG was at least 16% higher among piglets from HCA-treated sows than piglets from CON sows in the 21-day post-wean phase (Figure 5b).

### 3.5. Prenatal Stress Effects on Cortisol and Immune to ACTH Challenge

**Figure 6:**
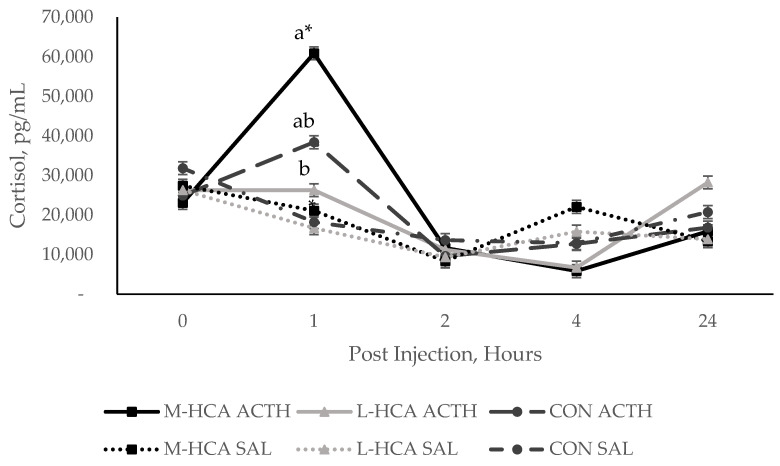
Pig plasma cortisol concentrations at various time points post-ACTH or saline injections at 21 days post-wean, n = 24. Injection type was adrenocorticotropic hormone (ACTH) or saline (SAL). Sow treatments were hydrocortisone acetate (HCA) capsules or placebo (CON) fed for 21 days during mid-gestation (M-HCA) or late gestation (L-HCA). Data are expressed as means ± standard error of the mean. Means denoted with a different alphabetical superscript differ at *p* < 0.05 across sow treatment but within injection type, means denoted with * differ at *p*-value < 0.05 within sow treatment but across injection type according to the Tukey–Kramer adjustment. Treatment × Injection Type × Hour *p* < 0.01.

Twenty-one days post-weaning, pigs were challenged with ACTH or saline to further observe maternal treatment effects on HPA responsiveness over 24 h. Similarly to weaning stress, injection with ACTH resulted in differing responses of piglets from different sow treatments (*p*-value < 0.01), with pigs from M-HCA-treated sows having the highest plasma cortisol response at 1 h post-injection. Specifically, piglets of M-HCA-treated sows injected with ACTH had cortisol concentrations 79%, 45% and 97% higher than ACTH-injected piglets from L-HCA-treated, CON sows and saline-injected piglets from M-HCA-treated sows, respectively (Figure 6). Notably, piglets from L-HCA-treated sows injected with ACTH exhibited the lowest cortisol concentration. They did not noticeably differ from their saline-injected counterparts. Moreover, piglets from M-HCA-treated sows had lower overall cortisol-binding globulin (CBG) levels (*p*-value = 0.04).

In contrast, those from L-HCA-treated sows exhibited the highest (Table 5) and were the only ones with significantly different CBG levels at 1 h and 24 h post-injection regardless of receiving the ACTH or saline injection. No other stress biomarkers tested were found to differ significantly based on maternal treatment (Table 5).

Both sow treatment and injection type affected leukocyte populations, specifically the percentage of neutrophils (*p*-value = 0.06), lymphocytes (*p*-value = 0.05), and the neutrophil-to-lymphocyte ratio (*p*-value = 0.05), with piglets from L-HCA-treated sows that received the ACTH or saline injection not differing in their percentages (Table 6). Meanwhile, piglets from M-HCA-treated and CON sows were significantly different based on injection type for all three leukocyte measures, with those that received the ACTH injection having lower neutrophil percentages and higher lymphocyte percentages, resulting in overall lower N:L ratios (Table 6).

Mitogen-induced lymphocyte proliferation showed a similar response to ACTH challenge as seen in response to weaning stress, with sow treatment tending to have an overall effect (*p*-value = 0.06). Piglets of CON sows exhibited the lowest T-cell proliferation, being significantly lower than that of piglets from M-HCA-treated sows (0.84 vs. 1.69 proliferation indices, respectively) while also being lower, though not significantly, than that of piglets from L-HCA-treated sows (L-HCA = 1.12 proliferation index). B-cell proliferation did not differ by maternal treatment (*p*-value = 0.30).

## 4. Discussion

Maternal stress during gestation can affect the development and responsiveness of the hypothalamic–pituitary–adrenal axis (HPA) and immune system in the progeny. Gestational stage, duration, and type of stressor may also influence the short- and long-term effects on the future progeny. This study revealed differential effects of hydrocortisone treatment (HCA) and days of gestation at which HCA was fed on the immune phenotype and stress biomarkers in the progeny. Consequences of maternal stress occurred at birth, during the suckling and post-wean phases, and in response to an adrenocorticotropic hormone (ACTH) challenge. Piglets born to sows fed HCA during mid-gestation were observed to have reduced measures of humoral immunity, indicated by changes in immunoglobulins and cytokines associated with the humoral immune response, whereas piglets born to sows administered HCA in late gestation were found to have a more robust stress response to farrowing but a dampened or delayed stress response to weaning and ACTH challenge, as evidenced by changes in cortisol and pro-inflammatory cytokine concentrations, leukocyte populations, and stereotypic behavior. The differences in measures of immune and stress between piglets born to sows stressed during mid and late gestation reported within imply that maternal stress and the stage of gestation at which stress occurs can differentially affect immune phenotype and stress responsiveness of the progeny during lactation and 21 days post-weaning.

In utero exposure to elevated maternal cortisol during gestation may be partly attributed to the different immune phenotypes observed at birth and throughout lactation in the progeny. Although the data presented solely reflect the expression of the progeny, combining these results with the lack of difference in colostral cortisol across treatment may indicate that in utero exposure to elevated cortisol levels may have influenced these differences. In utero, exposure to high cortisol levels can impact the development and functionality of physiological systems, including the HPA axis, which undergoes further development later in gestation [4,24]. This may partly explain why some aspects of the immune response did not differ regardless of when chronic maternal stress occurred and may account for the higher levels of stress markers like cortisol and neutrophil-to-lymphocyte ratio in the umbilical cord blood of piglets born to late-stressed sows and that remained elevated throughout lactation. However, it is also plausible that these may reflect a more robust stress response to farrowing in the sows. The blood collected from the umbilical cord was a mixture of venous and arterial, indicating that the blood sample was representative of both the sow and her progeny.

It has been established during pregnancy that maternal cortisol remains constant until parturition [25]. Despite the cortisol increase before the initiation of parturition, changes in the phenotype of the progeny born to sows in this study were absent, suggesting a protective mechanism that controls cortisol levels during pregnancy, especially during vulnerable periods, such as early and mid-gestation, to maintain pregnancy [26,27]. One possible mechanism that regulates maternal cortisol is the enzyme 11β-Hydroxysteroid dehydrogenase (11β-HSD2). It has been shown to control cortisol levels during gestational stress in humans, mice, and pigs [28,29]. In humans, it was found that lower levels of the 11β-HSD2 enzyme on the placental side resulted in higher cortisol reactivity in children, which may be due to hindering the ability of the 11β-HSD2 enzyme to control maternal cortisol, thus exposing the developing progeny to elevated maternal cortisol [30]. Reduced placental expression of the enzyme may be associated with excess cortisol exposure, reducing the 11β-HSD2 enzyme’s capacity to convert cortisol to cortisone. Suppose maternal cortisol levels surpass the enzyme’s threshold; in this case, the enzyme’s protective effectiveness is reduced, which could increase the risk of fetal exposure.

Another possible explanation for the observed differences in progeny immune and stress responses could be the increasing levels of progesterone that occur as pregnancy progresses. Progesterone is associated with the retention of pregnancy via the stimulation of Th2 cytokines and the suppression of Th1 cytokines, which can result in pregnancy loss [31]. Interestingly, it has been noted that progesterone, similar to cortisol, is known to interact and can bind to corticosteroid-binding globulin (CBG). Cortisol-binding capacity may differ by stage of gestation [32,33], and progesterone competes with cortisol for binding to CBG [34]. This may partly explain differences. However, progesterone binds at a lower affinity than cortisol; thus, an increase in free cortisol compared to bound may expose the fetus to excess maternal cortisol. Therefore, if progesterone is bound, this would reduce concentration, which may be attributed to shifts in the Th1/Th2 balance during vulnerable periods of fetal development. This shift might affect fetal exposure to Th1 cytokines, potentially resulting in alterations in immune and stress responsiveness observed at birth and during the suckling period. However, this study did not measure progesterone, so its role remains unknown.

Immunoglobulins do not passively cross the placenta; thus, pigs obtain passive immunity via colostrum consumption [21]. Immunoglobulin-G (IgG) is the most common antibody for swine passive immunity [35], with immunoglobulin-A (IgA) being secondary and playing a vital role in mucosal immunity [36]. Decreased immunoglobulin concentrations in colostrum samples of dams stressed during mid-gestation and decreased IgG and IgA levels in their progeny over the 21-day lactation period may indicate a dampened humoral response. While little work has explored the effect of maternal stress on the pig’s passive immunoglobulin levels, studies observed that IgA levels in milk were reduced in humans and mice exposed to psychological stress during late gestation [37,38]. While reduced immunoglobulin concentrations in the piglets may indicate reduced immunoglobulin levels in the colostrum or milk, it has been suggested that increased maternal cortisol at birth may accelerate the neonate’s gut closure in both piglets and calves, which can limit maternal antibody absorption [39,40]. However, it is unlikely that decreased immunoglobulin concentrations are due to gut closure. However, initial immunoglobulin differences may be due to alteration during HCA treatment in the dam.

The offspring of mid-stressed sows may have a dampened humoral response. However, those born to late-stressed sows had a more enhanced measure of an immune response, particularly the adaptive immune system. The adaptive immune system, specifically its antibody-producing capacity, can take several weeks of life to mature in the pig [41]. Thus, measures of adaptive immunity before weaning likely reflect the influence that the sow and her colostrum and milk composition have on the response of her progeny. When comparing the offspring of sows fed HCA during late gestation to those from sows heat-stressed at the same stage of gestation, piglets of late heat-stressed sows exhibited a slower decline in IgG concentration over a 20-day lactation phase [20], similar to the piglets of late-stressed sows in the current study. This, in conjunction with patterns of IgG concentration in piglets of the current study, may indicate that late gestation stress affects the acquisition of passive immunity differently than mid-gestation stress.

Contrary to the reduced measures of humoral immunity, piglets from sows fed HCA during mid-gestation exhibited the highest B cell proliferation at 7 days of lactation, while those born to sows fed HCA during late gestation had higher T cell proliferation at 14 days of lactation and 14 days post-weaning. Couret et al. [11] similarly found that piglets born to sows that experienced social stress at late gestation had higher T and B cell proliferation at 19 days of age. However, others have found a reduction in the lymphocyte proliferative ability of piglets born to late gestationally stressed sows [16]. In non-human primates, it has been speculated that prenatal stress reduces the sensitivity of progeny’s lymphocytes to inhibition by cortisol [42,43], which may partly explain why reductions in proliferation were not seen in the piglets from treated sows in the current study in response to weaning stress and ACTH challenge. However, this may also be partially due to the delayed or dampened cortisol response in piglets of late-stressed sows.

Multiple studies have found that gestational stress that occurs in late gestation leads to differences in HPA axis organization in the progeny [4,10,44]. Alterations in mRNA associated with ACTH receptors were found in piglets of sows that experienced stress during late gestation [4,10], while Kanitz et al. [44] found a reduction in hypothalamic glucocorticosteroid receptor-binding sites, which may affect the negative feedback mechanism. These alterations may lead to differing physiological and behavioral responses to stressors, as observed in the current study, such as the delay in cortisol peak in response to weaning and the delayed or dampened cortisol response to ACTH challenge in pigs born to dams that received HCA in late gestation. Previous research found that piglets born to sows socially stressed in mid- or late gestation exhibited an elevated and sustained cortisol response to mixing stress [9], while Kranendonk et al. [12] found that piglets of sows that were administered HCA in late gestation had lower cortisol responses to ACTH injection than those of piglets from sows administered HCA in mid-gestation or control dams. There is some speculation that ACTH synthesis may be inhibited by monocyte-produced TNF-α [45], which is particularly interesting, as in the current study, we found piglets born to sows administered HCA in late gestation not only had higher TNF-α and monocyte percentages in response to weaning stress but also increased IL-17, a known recruiter and activator of monocytes [46,47]. Though measures of cytokines were not observed during the ACTH challenge, this may provide possible insight into the mechanism of altered stress responsiveness and should be studied further. In contrast, others have found that piglets born to sows exposed to social or restraint stress during late gestation had either reduced or similar pro-inflammatory cytokine levels [11,48], which may highlight the differing effect of maternal stressor type on alterations in the progeny.

Finally, maternal stressor type and gestational stage at occurrence may also influence progeny behavioral responses to stressors [3], which could be attributed to the alteration of ACTH receptors and hypothalamic glucocorticosteroid receptors. Though the current study did not find significant effects of maternal treatment or gestational stage on aggressive encounters, it is noteworthy that there was a decreased time spent in aggressive encounters and the likelihood of inciting or receiving aggressive encounters seen in the piglets of late-gestation HCA-treated sows. This observation aligns with the significant reduction in frequency and duration of observed stereotypic behaviors and a delay in cortisol peak in these piglets. Ison et al. [49] reported similar findings, with piglets born to sows socially stressed at mid-gestation exhibiting less aggression and overall activity in response to weaning than piglets from control sows. However, consistent with mixed results on cortisol responses, some have found that piglets born to sows administered HCA in mid and late gestation had fewer non-aggressive encounters than their counterparts from non-treated sows [12].

In contrast, others found that ACTH administration in late gestation resulted in no behavioral changes in response to mixing [14]. It is plausible that reductions in aggressive and stereotypic behavior seen in the piglets of late-gestation HCA-treated sows in the current study are correlated with the observed delay in cortisol response to the administered stressors. However, the linkage between gestational stress and physiological and behavioral stressor responses in the progeny seems to depend on maternal stressor type and gestational timing. While these findings likely support the speculation of in utero exposure playing a leading role in progeny HPA alteration, even less is known about the effect of abnormal maternal cortisol levels on the development and function of the progeny’s immune response. There is still a gap in understanding how maternal stress during gestation affects the offspring’s immune phenotype and function. These findings highlight that the timing of this stress critically shapes a piglet’s immune response, stress reactivity, and behavior.

## 5. Conclusions

The results of the present study reveal that exogenous oral administration of synthetic glucocorticoid hydrocortisone acetate for 21 days to pregnant sows differentially impacts the stress axis and immune development of the progeny at birth and up to 21 days post-weaning. More specifically, we conclude that elevated circulating cortisol during mid-gestation may initially affect passive immunity, as revealed by changes in immunoglobulins and cytokines associated with humoral immunity. At the same time, elevated cortisol during late gestation may have a greater effect on the development of the stress axis, as these piglets had a dampened or delayed stress response to weaning and ACTH, with delays in stress-related measures such as cortisol and pro-inflammatory cytokines and decreased stress-associated behaviors. Further studies are required to elucidate directly the impact of these differential phenotypes in the progeny in the face of stressors and immunological challenges to determine the overall impact on health and well-being.

## Figures and Tables

**Table 1:** Immunoglobulins, cortisol, and cytokine concentrations in colostrum samples collected at the onset of farrowing from sows fed hydrocortisone acetate or placebo for 21 days during mid- or late gestation.

Measures ^1^	M-HCA ^2^	L-HCA ^2^	CON ^2^	*p*-Value
Immunoglobulin A, ng/mL	15.32 ± 0.11 ^a1^	15.70 ± 0.11 ^ab2^	16.00 ± 0.11 ^b^	0.01
Immunoglobulin G, ng/mL	19.04 ± 0.34	19.45 ± 0.34	18.84 ± 0.34	0.48
Immunoglobulin E, ng/mL	0.00 ± 5.23	9.05 ± 5.23	0.00 ± 5.23	0.42
Immunoglobulin M, ng/mL	15.05 ± 0.31	15.02 ± 0.31	14.68 ± 0.31	0.66
Cortisol, pg/mL	10.97 ± 0.06	10.96 ± 0.07	10.97 ± 0.06	0.99
Interleukin-10, pg/mL	2.39 ± 0.89	2.34 ± 0.89	2.42 ± 1.09	1.00
Interleukin-4, pg/mL	1.97 ± 1.45	3.68 ± 1.45	6.90 ± 1.78	0.19
Interleukin-17, pg/mL	1.72 ± 1.95	2.04 ± 1.95	2.69 ± 2.39	0.95

^1^ Data are expressed as log-transformed means ± standard error of the mean; except for IgE, which is expressed as non-log-transformed), n = 10. ^2^ Sow treatments were hydrocortisone acetate (HCA) capsules or placebo (CON) fed for 21 days during mid-gestation (M-HCA) or late gestation (L-HCA). ^a,b^ Means with different superscripts differ at *p*-value < 0.05, and those with ^1,2^ superscripts differ at 0.05 < *p*-value < 0.10 according to the Tukey–Kramer adjustment.

**Table 2:** Effects of maternal treatment on mean percentages of neutrophils and lymphocytes and concentrations of immunoglobulins, cytokines, and other stress biomarkers in umbilical cord blood from piglets born to these sows.

Measures	M-HCA ^3^	L-HCA ^3^	CON ^3^	*p*-Value
Neutrophil, % ^1^	14.88 ± 4.24 ^a1^	34.11 ± 3.00 ^b^	27.21 ± 2.56 ^ab2^	<0.01
Lymphocyte, % ^1^	77.98 ± 4.96 ^a1^	57.54 ± 3.51 ^b^	63.95 ± 2.99 ^ab2^	0.01
Neutrophil to lymphocyte ratio ^1^	0.19 ± 0.12 ^a^	0.66 ± 0.08 ^b^	0.45 ± 0.07 ^ab^	0.01
Immunoglobulin-G, ng/mL ^2^	12.13 ± 0.75	11.93 ± 0.62	12.51 ± 0.67	0.82
Immunoglobulin-A, ng/mL ^2^	9.68 ± 0.33	9.90 ± 0.27	10.46 ± 0.29	0.22
Interleukin-10, pg/mL ^1^	7.11 ± 1.34	7.31 ± 1.09	7.39 ± 1.20	0.99
Tumor necrosis factor-α, pg/mL ^1^	156.01 ± 93.4	64.99 ± 76.26	0	0.48
Interferon-ɣ, pg/mL ^1^	1.78 ± 1.94	3.19 ± 1.94	1.46 ± 1.59	0.79
Cortisol, pg/mL ^2^	10.97 ± 0.16 ^ab^	11.25 ± 0.13 ^b^	10.59 ± 0.15 ^a^	0.02
Corticotropin-releasing hormone, pg/mL ^1^	41.61 ± 22.46	45.97 ± 10.05	31.96 ± 11.23	0.66
Adrenocorticotropic hormone, pg/mL ^1^	7.68 ± 10.63	14.59 ± 4.76	11.17 ± 5.32	0.80
11β-Hydroxysteroid dehydrogenase-2, ng/mL ^1^	38.56 ± 6.74	45.70 ± 6.03	39.60 ± 6.03	0.69

^1^ Data are expressed as means ± the standard error of the mean (n = 36). ^2^ Data are expressed as log-transformed means ± standard error of the mean (n = 36). ^3^ Sow treatments were hydrocortisone acetate (HCA) capsules or placebo (CON) fed for 21 days during mid-gestation (M-HCA) or late gestation (L-HCA). ^a,b^ Means with different superscripts differ at *p*-value < 0.05, and those with ^1,2^ superscripts differ at 0.05 < *p*-value < 0.10 according to the Tukey–Kramer adjustment.

**Table 3:** Effect of maternal treatment on mean percentages of neutrophils and lymphocytes, neutrophil-to-lymphocyte ratio and immunoglobulin-A concentrations for piglets over a 21-day lactation period.

Measures ^1^	M-HCA ^3^	L-HCA ^3^	CON ^3^	*p*-Value
Neutrophil, %	38 ± 2.0 ^1^	44 ± 2.0 ^2^	40 ± 2.0 ^12^	0.09
Lymphocyte, %	55 ± 2.0	49 ± 2.0	52 ± 2.0	0.17
Neutrophil to lymphocyte ratio	0.77 ± 0.09 ^1^	1.06 ± 0.09 ^2^	0.91 ± 0.09 ^12^	0.09
Immunoglobulin-A, ng/mL ^2^	12.19 ± 0.13 ^1^	12.45 ± 0.13 ^12^	12.63 ± 0.13 ^2^	0.08

^1^ Data are expressed as means ± the standard error of the mean, n = 36. ^2^ Data are expressed as log-transformed means ± standard error of the mean. ^3^ Sow treatments were hydrocortisone acetate (HCA) capsules or placebo (CON) fed for 21 days during mid-gestation (M-HCA) or late gestation (L-HCA). ^12^ Means with different superscripts differ at 0.05 < *p*-value < 0.10 according to the Tukey–Kramer adjustment.

**Table 4:** Effect of maternal treatment on aggressive encounters and oral-nasal-facial (ONF) stereotypes in the progeny ^1,2,3^.

Measures	M-HCA	L-HCA	CON	SEM	*p*-Value
Initiator of aggressive encounter, no.	12.60	11.60	16.60	4.52	0.77
Receiver of aggressive encounter, no.	13.20	12.00	16.80	4.35	0.73
Total number of aggressive encounters	25.80	24.60	33.40	8.53	0.74
Total duration of aggressive encounters, sec	1149.20	772.80	1392.20	378.03	0.52
Total number of oral-nasal-facial events	10.20 ^a^	2.60 ^b1^	9.40 ^ab2^	2.04	0.04
Total duration of oral-nasal-facial events, sec	176.00 ^1^	49.20 ^2^	164.00	37.87	0.07

^1^ Data are expressed as means ± standard error of the mean, n = 36. ^2^ Means that differ at *p*-value < 0.05 are denoted by differing alphabetical superscripts and means that differ at 0.05 < *p*-value < 0.10 according to the Tukey–Kramer adjustment are denoted by differing numerical superscripts. ^3^ Sow treatments were hydrocortisone acetate (HCA) capsules or placebo (CON) fed for 21 days during mid-gestation (M-HCA) or late gestation (L-HCA).

**Table 5:** Effect of maternal treatment on stress biomarkers in piglets at 1, 2, 4, and 24 h post-ACTH challenge ^1,2,3^.

Measures	M-HCA	L-HCA	CON	*p*-Value
CRH, pg/mL				0.34
1 h	58.00 ± 6.79	43.59 ± 5.99	66.95 ± 5.54	
2 h	47.07 ± 5.54	47.41 ± 5.99	47.68 ± 5.99	
4 h	28.37 ± 5.99	34.63 ± 5.99	39.34 ± 5.99	
24 h	28.65 ± 5.99	34.64 ± 5.99	32.62 ± 6.79	
ACTH, pg/mL				0.22
1 h	1.30 ± 6.28	8.39 ± 5.92	0.00	
2 h	8.53 ± 5.13	9.34 ± 5.54	0.00	
4 h	5.11 ± 5.54	8.24 ± 5.54	4.92 ± 5.54	
24 h	0.05 ± 5.54	9.46 ± 5.54	2.42 ± 6.28	
11β-HSD2, ng/mL				0.25
1 h	133.45 ± 20.30	200.48 ± 20.30	194.74 ± 18.80	
2 h	141.69 ± 18.80	157.56 ± 20.30	143.09 ± 21.70	
4 h	83.85 ± 20.30	71.03 ± 20.30	65.76 ± 20.30	
24 h	153.98 ± 18.80	158.53 ± 20.30	114.99 ± 24.27	
CBG, ng/mL				0.043
1 h	177.23 ± 35.06 ^a^	399.77 ± 35.06 ^b1^	300.60 ± 32.46 ^ab^	
2 h	268.95 ± 32.46	314.36 ± 35.06	315.97 ± 35.06	
4 h	215.59 ± 35.06	241.80 ± 35.06	226.04 ± 35.06	
24 h	223.64 ± 32.46	217.12 ± 35.06 ^2^	187.79 ± 41.91	

^1^ Data are expressed as means ± standard error of the mean (n = 24). ^2^ Means that differ at *p*-value < 0.05 across sow treatment but within an hour according to the Tukey–Kramer adjustment are denoted with a different alphabetical superscript; means that differ at *p*-value < 0.05 within sow treatment but across an hour according to the Tukey–Kramer adjustment are denoted with a different numerical superscript. ^3^ Sow treatments were hydrocortisone acetate (HCA) capsules or placebo (CON) fed for 21 days during mid-gestation (M-HCA) or late gestation (L-HCA).

**Table 6:** Interactive effect of maternal treatment and ACTH challenge on mean percentages of neutrophils and lymphocytes and neutrophil-to-lymphocyte ratio in the progeny ^1,2,3,4^.

	M-HCA		L-HCA		CON		*p*-Value
Measures	ACTH	SAL	ACTH	SAL	ACTH	SAL	
Neutrophil, %	33.04 ± 2.40 *	44.16 ± 2.32 *	37.14 ± 2.40	37.75 ± 2.40	29.49 ± 2.40 *	39.75 ± 2.47 *	0.06
Lymphocyte, %	57.90 ± 2.30 *	45.38 ± 2.23 *	52.84 ± 2.30	50.95 ± 2.30	59.93 ± 2.30 *	48.68 ± 2.37 *	0.05
Neutrophil-to-lymphocyte ratio	0.65 ± 0.10 *	1.09 ± 0.09 *	0.77 ± 0.10	0.78 ± 0.10	0.51 ± 0.10 *	0.91 ± 0.10 *	0.05

^1^ Data are expressed as means ± standard error of the mean (n = 24). ^2^ Means that differ at *p* < 0.05 within sow treatment but across injection type according to the Tukey–Kramer adjustment are denoted with an *. ^3^ Sow treatments were hydrocortisone acetate (HCA) capsules or placebo (CON) fed for 21 days during mid-gestation (M-HCA) or late gestation (L-HCA). ^4^ Injection types were ACTH (adrenocorticotropic hormone) and SAL (saline).

## Data Availability

Data presented in this study are available upon request from the corresponding author.

## References

[1] Graham A.M., Rasmussen J.M., Entringer S., Ben Ward E., Rudolph M.D., Gilmore J.H., Styner M., Wadhwa P.D., Fair D.A., Buss C. (2019). Maternal Cortisol Concentrations during Pregnancy and Sex-Specific Associations With Neonatal Amygdala Connectivity and Emerging Internalizing Behaviors. Biol. Psychiatry.

[2] Merlot E., Couret D., Otten W. (2008). Prenatal Stress, Fetal Imprinting and Immunity. Brain Behav. Immun..

[3] Otten W., Kanitz E., Tuchscherer M. (2015). The Impact of Prenatal Stress on Offspring Development in Pigs. J. Agric. Sci..

[4] Schwerin M., Kanitz E., Tuchscherer M., Brüssow K.-P., Nürnberg G., Otten W. (2005). Stress-Related Gene Expression in Brain and Adrenal Gland of Porcine Fetuses and Neonates. Theriogenology.

[5] Otten W., Kanitz E., Tuchscherer M., Schneider F., Brüssow K.-P. (2004). Effects of Adrenocorticotropin Stimulation on Cortisol Dynamics of Pregnant Gilts and Their Fetuses: Implications for Prenatal Stress Studies. Theriogenology.

[6] Otten W., Kanitz E., Couret D., Veissier I., Prunier A., Merlot E. (2010). Maternal Social Stress during Late Pregnancy Affects Hypothalamic-Pituitary-Adrenal Function and Brain Neurotransmitter Systems in Pig Offspring. Domest. Anim. Endocrinol..

[7] Nugent B.M., Bale T.L. (2015). The omniscient placenta: Metabolic and epigenetic regulation of fetal programming. Front. Neuroendocrinol..

[8] Merlot E., Quesnel H., Prunier A. (2013). Prenatal stress, immunity and neonatal health in farm animal species. Animal.

[9] Jarvis S., Moinard C., Robson S.K., Baxter E., Ormandy E., Douglas A.J., Seckl J.R., Russell J.A., Lawrence A.B. (2006). Programming the Offspring of the Pig by Prenatal Social Stress: Neuroendocrine Activity and Behaviour. Horm. Behav..

[10] Haussmann M.F., Carroll J.A., Weesner G.D., Daniels M.J., Matteri R.L., Lay D.C. (2000). Administration of ACTH to Restrained, Pregnant Sows Alters Their Pigs’ Hypothalamic-Pituitary-Adrenal (HPA) Axis2. J. Anim. Sci..

[11] Couret D., Prunier A., Mounier A.-M., Thomas F., Oswald I.P., Merlot E. (2009). Comparative Effects of a Prenatal Stress Occurring during Early or Late Gestation on Pig Immune Response. Physiol. Behav..

[12] Kranendonk G., Hopster H., Fillerup M., Ekkel E.D., Mulder E.J., Taverne M.A. (2006). Cortisol Administration to Pregnant Sows Affects Novelty-Induced Locomotion, Aggressive Behaviour, and Blunts Gender Differences in Their Offspring. Horm. Behav..

[13] Otten W., Kanitz E., Tuchscherer M., Puppe B., Nürnberg G. (2007). Repeated Administrations of Adrenocorticotropic Hormone during Gestation in Gilts: Effects on Growth, Behaviour and Immune Responses of Their Piglets. Livest. Sci..

[14] Lay D.C., Kattesh H.G., Cunnick J.E., Daniels M.J., Kranendonk G., McMunn K.A., Toscano M.J., Roberts M.P. (2011). Effect of Prenatal Stress on Subsequent Response to Mixing Stress and a Lipopolysaccharide Challenge in Pigs1. J. Anim. Sci..

[15] Sinkora M., Butler J.E. (2009). The ontogeny of the porcine immune system. Dev. Comp. Immunol..

[16] Tuchscherer M., Kanitz E., Otten W., Tuchscherer A. (2002). Effects of Prenatal Stress on Cellular and Humoral Immune Responses in Neonatal Pigs. Vet. Immunol. Immunopathol..

[17] Wang W., Sung N., Gilman-Sachs A., Kwak-Kim J. (2020). T Helper (Th) Cell Profiles in Pregnancy and Recurrent Pregnancy Losses: Th1/Th2/Th9/Th17/Th22/Tfh Cells. Front. Immunol..

[18] Salak-Johnson J.L., McGlone J.J. (2007). Making Sense of Apparently Conflicting Data: Stress and Immunity in Swine and Cattle. J. Anim. Sci..

[19] Sabic D., Koenig J.M. (2020). A Perfect Storm: Fetal Inflammation and the Developing Immune System. Pediatr. Res..

[20] Machado-Neto R., Graves C.N., Curtis S.E. (1987). Immunoglobulins in Piglets from Sows Heat Stressed Prepartum. J. Anim. Sci..

[21] Borghesi J., Mario L.C., Rodrigues M.N., Favaron P.O., Miglino M.A. (2014). Immunoglobulin Transport during Gestation in Domestic Animals and Humans: A Review. Open J. Anim. Sci..

[22] Kranendonk G., Hopster H., van-Eerdenburg F., van Reenen K., Fillerup M., de Groot J., Korte M., Taverne M. (2005). Evaluation of oral administration of cortisol as a model for prenatal stress in pregnant sows. Am. J. Vet. Res..

[23] National Research Council (2012). Nutrient Requirements of Swine.

[24] Pond W.G., Boleman S.L., Fiorotto M.L., Ho H., Knabe D.A., Mersmann H.J., Savell J.W., Su D.R. (2000). Perinatal Ontogeny of Brain Growth in the Domestic Pig. Proc. Soc. Exp. Biol. Med..

[25] Entringer S., Buss C., Rasmussen J.M., Lindsay K., Gillen D.L., Cooper D.M., Wadhwa P.D. (2017). Maternal Cortisol During Pregnancy and Infant Adiposity: A Prospective Investigation. J. Clin. Endocrinol. Metab..

[26] Edwards R., Omtvedt I.T., Turman E.J., Rule D.R., Stephens D.F., Mahoney G.W.A. (1968). Reproductive Performance of Gilts Following Heat Stress Prior to Breeding and in Early Gestation. J. Anim. Sci..

[27] Omtvedt I.T., Nelson R.E., Edwards R.L., Stephens D.F., Turman E.J. (1971). Influence of Heat Stress During Early, Mid and Late Pregnancy of Gilts. J. Anim. Sci..

[28] Salvante K.G., Milano K., Kliman H.J., Nepomnaschy P.A. (2017). Placental 11 β-hydroxysteroid dehydrogenase type 2 (11β-HSD2) expression very early during human pregnancy. J. Dev. Orig. Health. Dis..

[29] Klemcke H.G., Christenson R.K. (1996). Porcine Placental 11β-Hydroxysteroid Dehydrogenase Activity. Biol. Reprod..

[30] Jahnke J.R., Terán E., Murgueitio F., Cabrera H., Thompson A.L. (2021). Maternal stress, placental 11β-hydroxysteroid dehydrogenase type 2, and infant HPA axis development in humans: Psychosocial and physiological pathways. Placenta.

[31] Kumar P., Magon N. (2012). Hormones in Pregnancy. Niger. Med. J..

[32] Oakey R.E. (1975). Serum Cortisol Binding Capacity and Cortisol Concentration in the Pregnant Baboon and Its Fetus during Gestation. Endocrinology.

[33] Nenke M.A., Zeng A., Meyer E.J., Lewis J.G., Rankin W., Johnston J., Kireta S., Jesudason S., Torpy D.J. (2017). Differential Effects of Estrogen on Corticosteroid-Binding Globulin Forms Suggests Reduced Cleavage in Pregnancy. J. Endocrinol. Soc..

[34] Gardill B.R., Vogl M.R., Lin H.-Y., Hammond G.L., Muller Y.A. (2012). Corticosteroid-Binding Globulin: Structure-Function Implications from Species Differences. PLoS ONE.

[35] Curtis J., Bourne F.J. (1971). Immunoglobulin Quantitation in Sow Serum, Colostrum and Milk and the Serum of Young Pigs. Biochim. Biophys. Acta.

[36] Wines B.D., Hogarth P.M. (2006). IgA Receptors in Health and Disease. Tissue Antigens.

[37] Kohanski K., Redmond S.B. (2017). The Effects of Psychological and Physical Stressors on the Secretion of Immunoglobulin A in Humans and Mice. Bios.

[38] Jarillo-Luna A., Rivera-Aguilar V., Garfias H.R., Lara-Padilla E., Kormanovsky A., Campos-Rodríguez R. (2007). Effect of Repeated Restraint Stress on the Levels of Intestinal IgA in Mice. Psychoneuroendocrinology.

[39] Bate L.A., Ireland W., Connell B.J., Grimmelt B. (1991). Development of the Small Intestine of Piglets in Response to Prenatal Elevation of Glucocorticoids. Histol. Histopathol..

[40] Osorio J.S. (2020). Gut Health, Stress, and Immunity in Neonatal Dairy Calves: The Host Side of Host-Pathogen Interactions. J. Anim. Sci. Biotechnol..

[41] Rooke J.A., Bland I.M. (2002). The Acquisition of Passive Immunity in the New-Born Piglet. Livest. Prod. Sci..

[42] Coe C.L., Lubach G.R. (2000). Prenatal Influences on Neuroimmune Set Points in Infancy. Ann. N. Y. Acad. Sci..

[43] Coe C.L., Kramer M., Kirschbaum C., Netter P., Fuchs E. (2002). Prenatal Stress Diminishes the Cytokine Response of Leukocytes to Endotoxin Stimulation in Juvenile Rhesus Monkeys. J. Clin. Endocrinol. Metab..

[44] Kanitz E., Otten W., Tuchscherer M., Manteuffel G. (2003). Effects of Prenatal Stress on Corticosteroid Receptors and Monoamine Concentrations in Limbic Areas of Suckling Piglets (Sus Scrofa) at Different Ages. J. Vet. Med. A Physiol. Pathol. Clin. Med..

[45] Jäättelä M., Ilvesmäki V., Voutilainen R., Stenman U.H., Saksela E. (1991). Tumor Necrosis Factor as a Potent Inhibitor of Adrenocorticotropin-Induced Cortisol Production and Steroidogenic P450 Enzyme Gene Expression in Cultured Human Fetal Adrenal Cells. Endocrinology.

[46] Mills K.H.G. (2023). IL-17 and IL-17-Producing Cells in Protection versus Pathology. Nat. Rev. Immunol..

[47] Shahrara S., Pickens S.R., Dorfleutner A., Pope R.M. (2009). IL-17 Induces Monocyte Migration in Rheumatoid Arthritis. J. Immunol..

[48] Collier C.T., Carroll J.A., Ballou M.A., Starkey J.D., Sparks J.C. (2011). Oral Administration of Saccharomyces Cerevisiae Boulardii Reduces Mortality Associated with Immune and Cortisol Responses to Escherichia Coli Endotoxin in Pigs. J. Anim. Sci..

[49] Ison S.H., D’Eath R.B., Robson S.K., Baxter E.M., Ormandy E., Douglas A.J., Russell J.A., Lawrence A.B., Jarvis S. (2010). ‘Subordination Style’ in Pigs? The Response of Pregnant Sows to Mixing Stress Affects Their Offspring’s Behaviour and Stress Reactivity. Appl. Anim. Behav. Sci..

